# Dual Adjuvant‐Loaded Peptide Antigen Self‐Assembly Potentiates Dendritic Cell‐Mediated Tumor Immunotherapy

**DOI:** 10.1002/advs.202403663

**Published:** 2024-07-29

**Authors:** Jaehyun Kim, Seyoung Kang, Jisu Kim, Seok‐Beom Yong, Shayan Fakhraei Lahiji, Yong‐Hee Kim

**Affiliations:** ^1^ Department of Bioengineering Institute for Bioengineering and Biopharmaceutical Research Hanyang University Seoul 04763 Republic of Korea; ^2^ Nucleic Acid Therapeutics Research Center Korea Research Institute of Bioscience and Biotechnology (KRIBB) Chungcheongbuk‐do 28116 Republic of Korea; ^3^ Cursus Bio Inc. Icure Tower Seoul 06170 Republic of Korea; ^4^ Institute for Bioengineering and Biopharmaceutical Research (IBBR) Hanyang University Seoul 04763 Republic of Korea

**Keywords:** dendritic cell immunotherapy, immune checkpoint blockade, self‐assembly, therapeutic cancer vaccine, tumor‐associated antigen

## Abstract

Clinical translation of current cancer vaccine research has been hampered by limited antitumor immune responses due to inefficient antigen delivery and presentation, suboptimal DC and T cell activation. Biomaterial‐based nanovaccine offers targeted antigen delivery, protection from degradation in vivo, and prolonged tumor therapeutic efficacy. This study introduces a lipid‐coated deoxycholic acid‐survivin nanoassembly (DA‐L‐DSA). Survivin, overexpressed in several cancer cells and involved in cancer cell growth and immune evasion, is selected as a tumor‐associated antigen. An major histocompatibility complex class I binding epitope of survivin is engineered into the nanoassembly. R848, TLR 7/8 agonist, and SD‐208, TGF‐beta receptor1 kinase inhibitor, are coencapsulated into the nanoassembly as potent adjuvants to boost DC maturation and enhance antigen presentation. The DA‐L‐DSA effectively stimulates the maturation of dendritic cells, migrates into lymph nodes, and enhances T‐cell activation and Th1 response. A substantial influx of cytotoxic T lymphocytes into primary tumors is observed in a murine melanoma model and demonstrates anti‐metastatic effects in a spontaneous breast cancer metastasis model. Furthermore, DA‐L‐DSA exhibits a remarkable synergistic effect in the combination therapy with immune checkpoint inhibitors alleviating immunosuppressive tumor microenvironment. Taken together, these findings suggest DA‐L‐DSA as a promising immuno‐therapeutic platform that could be applicable to diverse intractable cancers.

## Graphical abstract

This study demonstrates the efficacy of dendritic cell‐mediated anticancer immunotherapy in allograft melanoma models and metastatic breast cancer models by producing major histocompatibility complex (MHC) I binding peptide epitopes of survivin proteins expressed in various cancers in the form of self‐assembled nanostructures with dual adjuvants.

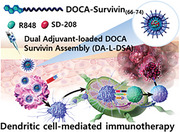

## 1 Introduction

Immunotherapy is a strategy in the fight against cancer, leveraging the immune system of the body to target and eradicate malignant cells.^[^ ^1^ ^]^ Central to the efficacy of cancer immunotherapy are dendritic cells (DCs), the paramount antigen‐presenting cells that bridge innate and adaptive immunity.^[^ ^2^ ^]^ The antigens presented on the DCs are recognized by the immune system. During this process, DCs migrate to lymph nodes, interact with naïve T cells, and modulate the activation of T cells, thereby controlling the immune response.^[^ ^3^ ^]^

Among the DC‐mediated cancer immunotherapies, the therapeutic cancer vaccine aims to improve treatment efficacy by enhancing immune responses against specific tumor antigens. Sipuleucel‐T, the first DC‐mediated vaccine, was approved by the US Food and Drug Administration (FDA) in 2010.^[^ ^4^ ^]^ For the vaccination, immature DCs are isolated from the patient's peripheral blood, matured, loaded with antigen, and infused back into the patient to induce a tumor‐specific immune response. However, The personalized nature of this vaccine, requiring ex vivo manipulation of patient‐derived DCs, introduces logistical challenges and potential for hypersensitivity reactions, underscoring the need for more universally applicable and effective strategies.^[^ ^5^ ^]^

The development of an effective therapeutic cancer vaccine necessitates overcoming several barriers. First, there is a need for a novel antigen delivery system that can effectively deliver antigens to DC and overcome insufficient antigen load.^[^ ^6^ ^]^ Second, cancer‐specific antigens should be recognized and delivered. Lastly, the immunosuppressive cells should be modulated for the recruitment of lymphocytes.^[^ ^7^ ^]^ Accordingly, the cancer vaccine should be designed to proficiently transport tumor‐associated antigen (TAA) to DCs, prompt DC maturation, leading to DC migration to the lymph nodes, and initiate activation of naïve T cells.^[^ ^8^ ^]^

Survivin, also referred to as a baculoviral inhibitor of apoptosis repeat‐containing 5 (BIRC5), is a member of the inhibitor of apoptosis protein family. Expression of survivin is used to regulate functions such as survival, growth, division, and angiogenesis.^[^ ^9^ ^]^ It is overexpressed in various cancers, such as melanoma and triple‐negative breast cancer, but remains barely apparent in normal cells.^[^ ^10^ ^]^ Accordingly, we have envisioned a therapeutic cancer vaccine intended to provide the survivin peptide epitope_(66‐74)_, which can be attached to major histocompatibility complex (MHC) class I of dendritic cells as a TAA‐derived peptide.^[^ ^10a^ ^]^ Moreover, we have devised a method for delivering R848 (resiquimod) and SD‐208 as adjuvants along with antigens to boost DC maturation and enhance antigen presentation. R848 is a compound with immune regulatory and antiviral properties that interact with Toll‐like receptor 7 (TLR7) and Toll‐like receptor 8 (TLR8). These Toll‐like receptors are pattern recognition receptors, detecting pathogens or abnormalities from the outside and regulating the immune response.^[^ ^11^ ^]^ In DCs, R848 activates TLR7 and TLR8, and enhances antigen expression and presentation.^[^ ^12^ ^]^ SD‐208 is a kinase inhibitor that targets TGF‐β receptor1 and prevents the downstream mechanisms that occur when TGF‐β is transmitted to cells.^[^ ^13^ ^]^ Previous studies have reported that TGF‐β inhibits dendritic cell maturation, antigen presentation, and T cell activation.^[^ ^14^ ^]^ In addition, deoxycholic acid can modulate the function of dendritic cells through the TGR5‐cAMP‐PKA pathway. Activation of TGR5 by deoxycholic acid inhibits the excessive activation of dendritic cells, leading to reduced excessive inflammatory responses. This suggests that deoxycholic acid could help in modulating the immune response, potentially making dendritic cells more tolerogenic, which could be useful in preventing excessive inflammation such as cytokine release syndrome during cancer treatment. Thus, we hypothesized that delivering survivin antigens, R848, and SD‐208 effectively to DC would effectively induce maturation and migration of DC to lymph nodes, resulting in an effective anti‐cancer immune response.

In this study, we synthesized a survivin peptide epitope monomer modified with deoxycholic acid to facilitate self‐assembly into structures with a hydrophobic core, enabling efficient encapsulation of R848 and SD‐208, and the assembled structure was encapsulated in a lipid membrane to facilitate cell penetration and stability in the body (dual adjuvants‐loaded, lipid‐coated deoxycholic acid survivin assembly; DA‐L‐DSA). DA‐L‐DSA administration markedly increased T cell infiltration within the tumor microenvironment, amplifying the anti‐tumor effects in synergy with immune checkpoint inhibitors. These findings position DA‐L‐DSA as a potent immunotherapeutic strategy against a spectrum of cancers, underscoring its potential clinical utility in melanoma and metastatic breast cancer treatment paradigms.

## 2 Results

### 2.1 Preparation and Characterization of DA‐L‐DSA

**Figure 1:**
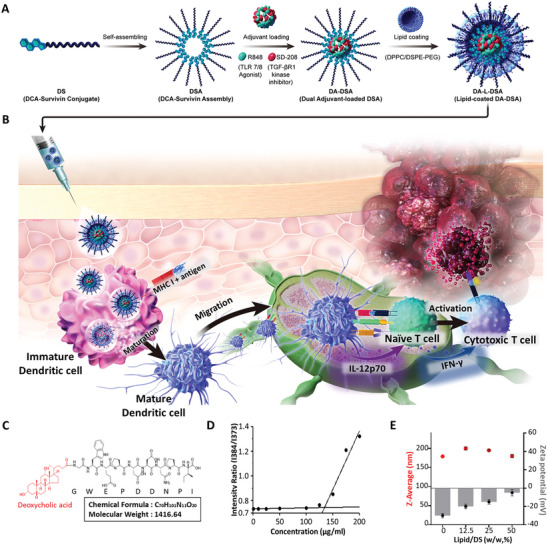
Fabrication method and mechanism of action of DA‐L‐DSA. A) Illustration of the synthetic procedure for dual adjuvant‐loaded, lipid‐coated deoxycholic acid‐survivin assembly (DA‐L‐DSA). The deoxycholic acid‐survivin (DS) was self‐assembled using an oil‐in‐water single emulsion technique in which R848 and SD‐208 were spontaneously encapsulated in the hydrophobic core. B) Subcutaneously injected DA‐L‐DSA was effectively delivered to dendritic cells to induce dendritic cell maturation and migrated to lymph nodes to induce antigen‐specific killing effect through activation of naive T cells. C) Chemical structure of DS conjugate. D) Determination of CMC of DS conjugate. The DS conjugate can form micelles at a concentration equal to or above133.25 µg mL^−1^. E) Hydrodynamic size and zeta potentials of L‐DSA. The size and zeta‐potentials were measured by dynamic light scattering. Data are expressed as the mean ± SD (*n* = 3).

To fabricate a nano self‐assembly system capable of delivering both a tumor‐associated antigen (TAA) and adjuvants, we conjugated the survivin peptide epitope_(66‐74)_, a TAA that binds to MHC class I on antigen‐presenting cells, with deoxycholic acid, yielding deoxycholic acid‐survivin (DS) (**Figure** **1**C and Figure S1, Supporting Information). Deoxycholic acid, consisting of hydrophilic moieties and a hydrophobic cyclopentanophenanthrene nucleus, enable deoxycholic acid to form micelles in water phase. Accordingly, It has been demonstrated that the relatively hydrophilic survivin peptide epitope is capable of forming a micelle structure when bound to deoxycholic acid, exhibiting amphiphilic properties (DSA). The amphiphilic DS peptide monomers can readily self‐assemble to form core‐shell structured peptide assembly, exhibiting a critical micelle concentration (CMC) of 133.25 µg mL^−1^ in PBS (pH 7.4) (Figure 1D). To induce a immune response, dual adjuvants (R848: TLR7/8 agonist and SD‐208: TGF‐βR1 kinase inhibitor) were loaded into the DSA (dual adjuvant‐loaded DSA; DA‐DSA). Then, DPPC/DSPE‐PEG‐based lipid membranes were coated on the surface of DA‐DSA (DA‐L‐DSA) (Figure 1A). Then, the DSA was coated with a lipid layer composed of DPPC: DSPE‐PEG_2000_ = 3:1 (L‐DSA). The hydrodynamic size and zeta potential were measured at the different weight ratios of the lipid and DS. The lipid weight percent to DS of 25% exhibited increases in zeta potential of −13.50 ± 2.40 mV and Z‐average size of 209.73 ± 1.04 nm, compared to those of DSA with −29.4 ± 1.20 mV and 177 ± 0.96 nm (Figure 1E).

To improve the immune response of L‐DSA, two adjuvants, R848 and SD‐208, were loaded in L‐DSA (DA‐L‐DSA). R848 activates immune cells through the TLR7/8 MyD88‐dependent signaling pathway, which induces interferon (IFN)‐α and interleukin (IL)−12. SD‐208 is an inhibitor of transforming growth factor β receptor (TGF‐βR) I kinase, which improves the dendritic cells, tumor‐specific cytotoxic T‐cell (CTL) responses, and also stimulates anti‐tumor natural killer cells. To investigate the synergistic effects of DS and two adjuvants on dendritic cells, immature dendritic cells were treated with antigen peptide and the adjuvants. At 24 h post‐treatment, the secretion of the immunostimulatory cytokine, IL‐12p70, and the expression of co‐stimulatory molecules CD80/CD86 were measured. CD80/CD86 and CD40 interact with CD28 and CD40L on T cells, respectively. The dendritic cells treated with the pre‐mixed antigen peptide and two adjuvants exhibited the highest maturation rate (Figure S2, Supporting Information).

**Figure 2:**
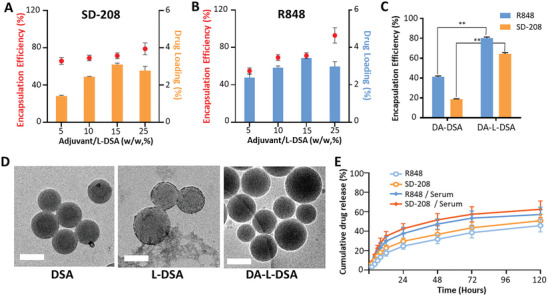
Physicochemical characterization of DA‐L‐DSA. A) Encapsulation efficiency and drug loading of SD‐208. B) Encapsulation efficiency and drug loading of R848. C) Enhancement of drug encapsulation efficiency with lipid‐coating. Statistical significance was calculated by Student's *t*‐test (*n* = 3), and *p*‐values were considered statistically significant (*p < 0.05, **p < 0.01, ***p < 0.001, ns = nonsignificant). D) Morphology of DSA, L‐DSA, and DA‐L‐DSA by transmission electron microscopy. Scale bars = 100 nm. E) Drug release profiles of DA‐L‐DSA measured in PBS with or without serum for 120 h by using a UV–vis spectrophotometer. All data are expressed as the mean ± SD (*n* = 3).

The drug loading and encapsulation efficiency of R848 and SD‐208 were measured at various weight percent of adjuvants to L‐DSA. Both adjuvants were loaded into L‐DSA and detected at different wavelengths, respectively. As a result, 15% of adjuvants to L‐DSA was found to be optimum for maximum loading of both adjuvants (**Figure** **2**A,B). Further improvement of drug encapsulation efficiency was achieved with lipid coating on the surface of the DSA. DA‐L‐DSA demonstrated higher encapsulation efficiencies than DA‐DSA by 1.8‐fold and 3.8‐fold for R848 and SD‐208, respectively (Figure 2C). The transmission electron microscope (TEM) image confirmed the presence of a lipid layer on the surface of L‐DSA compared to DSA. Furthermore, it was observed that the spherical shape remained even when adjuvants were loaded (Figure 2D).

The release profiles of R848 and SD‐208 were monitored in PBS (pH 7.4), and PBS containing 10% serum by replacing with fresh media at each time point. The DA‐L‐DSA demonstrated slightly faster release in serum‐containing media, as well as a sustained release pattern of two adjuvants without an initial burst. R848 and SD‐208 were released from the DA‐L‐DSA after 120 h at 37 °C by 57.19 ± 7.57% and 62.52 ± 8.71%, respectively in serum‐containing media (Figure 2E).

### 2.2 In Vitro Evaluation of Dendritic Cell Maturation with DA‐L‐DSA

**Figure 3:**
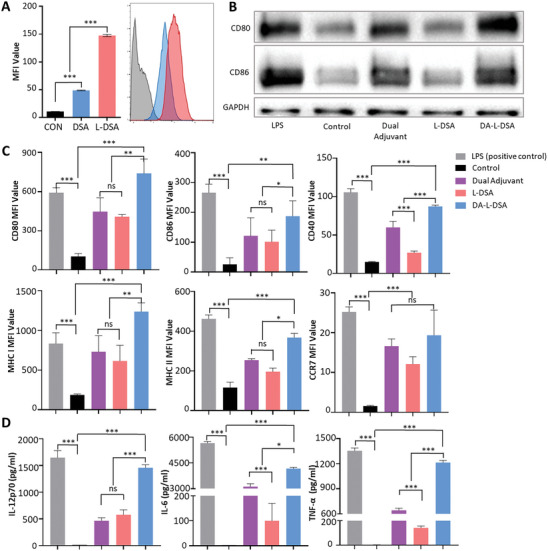
In vitro evaluation of bone‐marrow‐derived dendritic cell maturation with DA‐L‐DSA. A) The cellular uptake efficiency of DSA and L‐DSA for BMDC. Cells were analyzed by flow cytometry to measure the uptake of Cy5.5‐encapsulated DSA and L‐DSA. B) Western blot assay for BMDC activation. The expression of CD86, CD80, and GAPDH were detected by western blot. C) flow cytometric analysis of BMDC activation. The surface expression of CD80, CD86, CD40, MHC I, MHC II, and CCR7 were analyzed by flow cytometry. D) Enzyme‐linked immunosorbent assay (ELISA). The extracellular secretion of IL‐12p70, IL‐6, and TNF‐α were measured. Statistical significance was calculated by one‐way ANOVA with Tukey's post hoc test (*n* = 3), and *p*‐values were considered statistically significant (*p < 0.05, **p < 0.01, ***p < 0.001, ns = nonsignificant). All data are expressed as the mean ± SD (*n* = 3).

In order to determine the maximum non‐toxic concentration for further experiments, in vitro cytotoxicity of L‐DSA and DA‐L‐DSA was measured by CCK‐8 assay with bone marrow‐derived dendritic cells (BMDCs) incubated at various concentrations. Neither L‐DSA nor DA‐L‐DSA exhibited cytotoxicity at concentrations up to 100 × 10^−6^ m. To this end, the treatment concentration was set at 100 × 10^−6^ m for in vitro study (Figure S3, Supporting Information). To evaluate the contribution of the lipid membrane coated on the nano‐assembly in enhancing delivery efficiency into dendritic cells, BMDCs were treated with DSA and L‐DSA, both encapsulated with Cy5.5.The resulting data using flow cytometry demonstrated a higher MFI value with L‐DSA than DSA by 3.03‐fold (**Figure** **3**A).

To determine whether DA‐L‐DSA can mature dendritic cells effectively and induce T cell activation, BMDCs were treated for 24 h with DA‐L‐DSA for western blot and immunohistochemistry analysis. The CD80/86 expression in the DA‐L‐DSA‐treated group was found to be similar to that of the LPS‐treated group, which served as a positive control (Figure 3B and Figure S4, Supporting Information). In addition, flow cytometric analysis confirmed the significantly higher expression of CD80, CD86, CD40, MHC I, MHC II, and CCR7 in the DA‐L‐DSA‐treated group in comparison with the non‐treat group, only the adjuvants‐treated group, and the L‐DSA‐treated group (Figure 3C).

Furthermore, the DA‐L‐DSA‐treated group exhibited the highest levels of IL‐12p70, IL‐6, and TNF‐α release, known to activate naïve T cells, as detected by ELISA experiments, and showed almost the same degree as the LPS‐treated group, which is positive control (Figure 3D).

### 2.3 Delivery of L‐DSA to Lymph Nodes and Lymph Node‐Residing Dendritic Cells In Vivo

**Figure 4:**
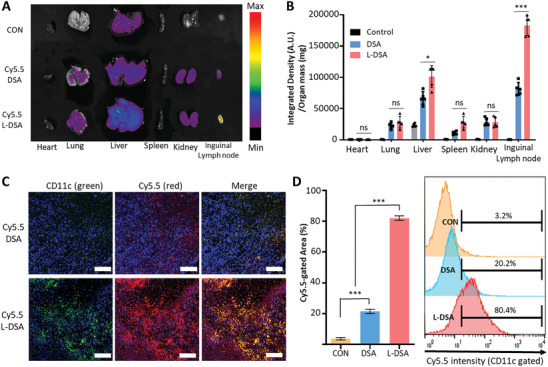
Intranodal and intracellular delivery of subcutaneously injected L‐DSA in vivo. A) Representative ex vivo organ images and B) Quantitative result of fluorescence signals in the major organs of mice at 24 h postinjection of Cy5.5‐loaded L‐DSA. Statistical significance was calculated by Student's t‐test (*n* = 3), and *p*‐values were considered statistically significant (*p < 0.05, **p < 0.01, ***p < 0.001, ns = nonsignificant). C) Representative immunofluorescence image showing the distribution of dendritic cells inside inguinal lymph nodes. Green = CD11c, Red = Cy5.5, Blue = DAPI. Scale bars = 200 µm. D) Distribution of dendritic cells present in lymph nodes confirmed by flow cytometry. Statistical significance was calculated by one‐way ANOVA with Tukey's post hoc test (*n* = 3), and *p*‐values were considered statistically significant (*p < 0.05, **p < 0.01, ***p < 0.001, ns = nonsignificant). All data are expressed as the mean ± SD (*n* = 3).

To evaluate the capability of DA‐L‐DSA in promoting an effective antitumor immune response, mice were administered subcutaneous injections of L‐DSA and DSA. Following a 24‐hour period, the biodistribution of Cy5.5‐labeled L‐DSA and DSA within the organism was investigated. We hypothesized that nano‐assemblies injected subcutaneously would undergo partial degradation, and some would be delivered to antigen‐presenting cells. Among these cells, dendritic cells would be activated to induce migration to lymph nodes. Indeed, L‐DSA showed significantly higher lymph node localization compared to DSA (**Figure** **4**A,B).

To confirm whether the observed signal was attributable to the migration of nano‐assembly‐delivered dendritic cells to the lymph nodes, the distribution of dendritic cells was assessed using immunofluorescence staining and flow cytometry. Consequently, the area where the dendritic cell marker CD11c and the fluorescent label Cy5.5 of the nano‐assemblies overlapped appeared yellow, representing the localization of nano‐assembly in the lymph node. The L‐DSA treatment group demonstrated a significantly brighter signal than the DSA treatment group (Figure 4C). Likewise, flow cytometry analysis of lymph nodes showed a significantly higher distribution of dendritic cells with Cy5.5 signals in the L‐DSA treatment group compared to the DSA treatment group or the non‐treatment group (Figure 4D).

### 2.4 Antitumor and Antimetastatic Efficacy of DA‐L‐DSA in Survivin‐Expressing Cancers

**Figure 5:**
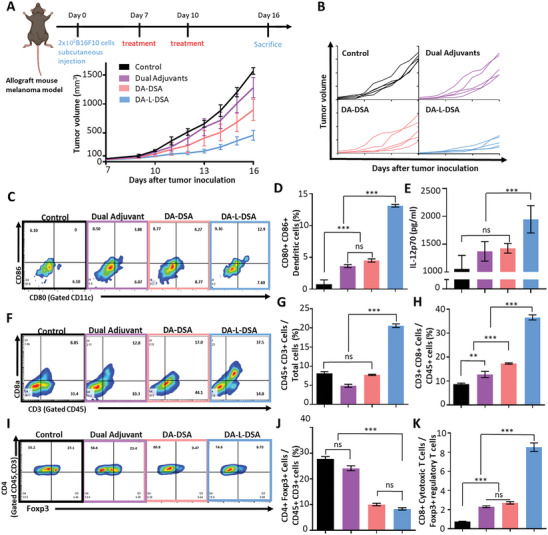
Antitumor immune response by DA‐L‐DSA in survivin‐expressing melanoma model. A) Schematic illustration of treatment schedules for verifying anti‐tumoral efficacies of DA‐L‐DSA and average growth profiles of tumors. Data are expressed as the mean ± SEM (*n* = 5). B) Growth profiles of individual tumors (*n* = 5). C,D) Flow cytometric analysis of dendritic cell maturation in inguinal lymph nodes. E) Quantification of IL‐12p70 inside the lymph nodes by ELISA. F–H) Flow cytometric analysis of CD3^+^ Tumor infiltrating lymphocytes, and CD8^+^ cytotoxic T cell recruitment in the primary tumors. I–K) Flow cytometric analysis of regulatory T cells in the primary tumors. Statistical significance was calculated by one‐way ANOVA with Tukey's post hoc test (*n* = 3), and *p*‐values were considered statistically significant (*p < 0.05, **p < 0.01, ***p < 0.001, ns = nonsignificant). All bar graph data are expressed as the mean ± SD (*n* = 3).

To evaluate the efficacy of DA‐L‐DSA in eliciting an antitumor immune response against survivin‐expressing cancers, an allograft murine model of melanoma was utilized. DA‐L‐DSA was administered subcutaneously to the mice on days 7 and 10 post‐tumor inoculation. All groups were sacrificed on day 16 and the primary tumor size was found to be 3.52‐fold smaller in the DA‐L‐DSA treatment group than in the non‐treat group (**Figure** **5**A,B).

To confirm the antitumor immune response by DA‐L‐DSA, lymph nodes were collected from mice, and flow cytometry and ELISA assays were performed to determine the degree of maturation and activation of dendritic cells distributed inside the lymph nodes. As a result of flow cytometry, it was confirmed that the population of activated dendritic cells in the lymph node was 4.64‐fold higher in the dual adjuvants‐treated group compared to the non‐treat group, 5.79‐fold higher in the DA‐DSA‐treated group, and 16.84‐fold higher in the DA‐L‐DSA‐treated group (Figure 5C,D). As a result of ELISA, it was also confirmed that IL‐12p70, a marker of T cell activation, derived from activated dendritic cells, was 1.84‐fold higher in the DA‐L‐DSA‐treated group compared to the non‐treat group (Figure 5E). In addition, primary tumors were obtained from mice for flow cytometric analysis, and immunohistochemistry assays to determine the degree of T cell infiltration and immunomodulation (Figure S6, Supporting Information). The flow cytometry confirmed immune activation induced by an increase in tumor‐infiltrating lymphocytes (TILs) into the tumor (Figure 5G). Among them, CD8^+^ T cells penetrated 4.21‐fold more than the non‐treat group (Figure 5F,H). Furthermore, the immunofluorescence staining images of the primary tumor also demonstrated that DA‐L‐DSA augmented the recruitment of CD8^+^ T cells into the tumor (Figure S7, Supporting Information). In addition, the results indicated that the ratio of regulatory T cells (Treg) was 3.33‐fold lower in the tumor of the DA‐L‐DSA‐treated group compared to the non‐treat group (Figure 5I,J). Overall, the ratio of CD8+ T cells to Tregs within the tumor microenvironment was significantly elevated in the DA‐L‐DSA group in comparison to other groups (Figure 5K).

**Figure 6:**
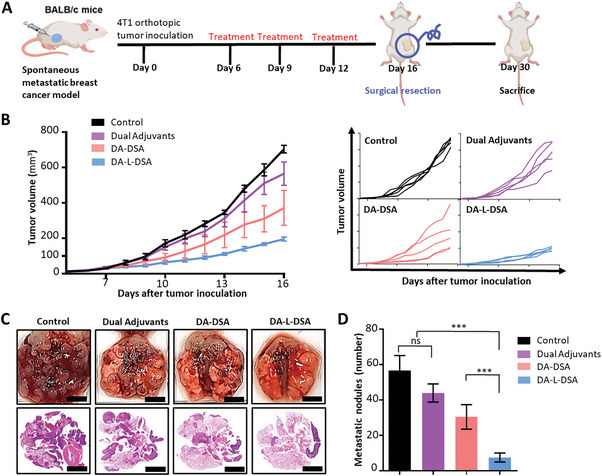
Antitumor and anti‐metastatic efficacy of DA‐L‐DSA in a spontaneous metastatic breast cancer model. A) Schematic illustration of treatment schedules for verifying anti‐tumoral and anti‐metastatic efficacies of DA‐L‐DSA. B) Average growth profiles of tumors and growth profiles of individual tumors. Data are expressed as the mean ± SEM (*n* = 5). C) Representative lung image and H&E staining image. Scale bars = 5 mm. D) Comparison of metastatic nodule number. Data are expressed as the mean ± SD (*n* = 5). Statistical significance was calculated by one‐way ANOVA with Tukey's post hoc test (*n* = 5), and *p*‐values were considered statistically significant (*p < 0.05, **p < 0.01, ***p < 0.001, ns = non‐significant).

To validate the effectiveness of DA‐L‐DSA in a metastatic cancer expressing survivin, a metastatic breast cancer model was established by injecting 4T1 cells into the 4th mammary gland of the Balb/c mouse. Following cancer cell inoculation, therapeutic cancer vaccines, DA‐L‐DSA were administered three times at three‐day intervals starting from day 6. On day 16, the tumor tissue was surgically removed and the degree of metastasis was evaluated by examining the lungs on day 30 (**Figure** **6**A).

In line with the results of the murine melanoma model, the primary tumor size of the DA‐L‐DSA‐treated group was 3.60‐fold smaller than that of the non‐treat group in the metastatic breast cancer model (Figure 6B and Figure S8, Supporting Information). In addition, experiments confirmed the anti‐metastatic effect of DA‐L‐DSA treatment. The non‐treatment group showed an average of 56.75 metastatic nodules, while the DA‐L‐DSA treatment group had 7.5 and 7.57‐fold fewer nodules (Figure 6C,D and Figure S9, Supporting Information).

### 2.5 Boosting Antitumor Efficacy of DA‐L‐DSA with Immune Checkpoint Inhibitor

**Figure 7:**
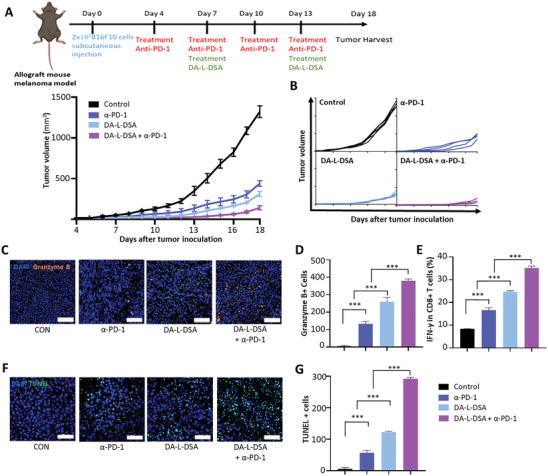
Synergistic anti‐tumor efficacy of DA‐L‐DSA with an immune checkpoint inhibitor. A) Schematic illustration of treatment schedules for verifying the synergistic effect of DA‐L‐DSA with conventional immune checkpoint blockade and average growth profiles of tumors. Data are expressed as the mean ± SEM (*n* = 5). B) Growth profiles of each tumor (n = 5). C) Representative immunofluorescence image of granzyme B^+^ cells in tumor. D) Quantification of granzyme B^+^ cells in the prior image. Scale bars = 100 µm. E) Flow cytometric analysis of the primary tumors for verifying T cell activation in tumor microenvironment. F) Representative TUNEL assay image. G) Quantification of TUNEL^+^ cells in the prior image. Scale bars = 50 µm. Statistical significance was calculated by one‐way ANOVA with Tukey's post hoc test (*n* = 3), and *p*‐values were considered statistically significant (*p < 0.05, **p < 0.01, ***p < 0.001, ns = non‐significant). All bar graph data are expressed as the mean ± SD (*n* = 3).

As the administration of DA‐L‐DSA was found to increase the influx of cytotoxic T cells and decrease Tregs in various cancers expressing survivin, it was further investigated whether the DA‐L‐DSA treatment can improve the response rate of immune checkpoint inhibitors by overcoming the limited response rate of existing immune checkpoint inhibitors. To this end, anti‐PD‐1 antibodies were administered to the allograft murine melanoma model every 3days from day 4 of tumor inoculation, and the DA‐L‐DSA was administered two times (**Figure** **7**A). Consequently, the combination therapy of DA‐L‐DSA and anti‐PD‐1 antibodies resulted in barely tumor growth (Figure 7B). The immunofluorescence staining results demonstrated a significant production of Granzyme B in the DA‐L‐DSA/ anti‐PD‐1 combinatory‐treated group, indicating activation of cytotoxic T cells within the tumor (Figure 7C,D). Flow cytometry results also indicated that the combination treatment of DA‐L‐DSA/ anti‐PD‐1 induced a 1.98‐fold higher IFN‐γ^+^CD8^+^ T cell population compared to the anti‐PD‐1 alone group and 1.7‐fold increase in the DA‐L‐DSA alone group, respectively. IFN‐γ secretion by CD8^+^ T cells assist effector responses and improves antitumor effects (Figure 7E and Figure S10, Supporting Information). The tumor cell apoptosis was verified through a TUNEL assay of the tumor tissues. Each DA‐L‐DSA, and anti‐PD‐1‐treated group induced apoptosis, and the combination treatment group showed the most abundant apoptotic tumor cells. These results verify that the combination treatment‐infiltrated CD8^+^ T cells have an anti‐tumoral property (Figure 7F,G).

## 3 Discussions

The advent of next‐generation gene sequencing technologies has significantly advanced our ability to predict tumor‐selective antigens, paving the way for the development of cancer immunotherapies that harness adaptive immune responses against tumor‐specific antigens. This approach marks a paradigm shift from traditional vaccines, focusing on therapeutic, rather than preventive, measures against cancer.^[^ ^15^ ^]^ The inherent diversity of cancer antigens poses challenges to preventive strategies; hence, the emphasis is on treating existing cancers by eliciting strong immune responses against cancer antigens, aiming to eradicate tumors and prevent their recurrence or metastasis.

For cancer vaccines to be effective, they must fulfill several criteria: antigens must be minimally expressed in normal cells, efficiently delivered to dendritic cells in substantial quantities, and capable of inducing a significant infiltration of cytotoxic T lymphocytes (CTLs) into the tumor microenvironment. The presence of CTLs not only exerts direct therapeutic effects but also enhances the efficacy of immune checkpoint blockades by amplifying the tumor‐killing capabilities of CTLs within the TME.^[^ ^16^ ^]^

Accordingly, to determine whether DA‐L‐DSA can mature dendritic cells effectively and induce T cell activation, BMDCs were treated with DA‐L‐DSA, and the expressions of the T cell co‐stimulatory molecules such as CD80, CD86, and CD40 on DCs were analyzed. CD80 and CD86 interact with CD28 and CD40 interacts with CD40L on T cells.^[^ ^17^ ^]^ The lipopolysaccharide (LPS)‐treated group was used as a positive control because LPS matures dendritic cells by activating TLR 4.^[^ ^18^ ^]^ The expression was significantly improved when the dendritic cells were incubated with DA‐L‐DSA compared to the control group after 24 h of treatment. In addition, the expression levels of MHC I and MHC II were increased, and the chemokine receptor CCR7, which is involved in lymph node migration by interacting with CCL19/CCL21 in the draining lymph node, was also upregulated.^[^ ^19^ ^]^ Similarly, secretion levels of immunostimulatory cytokines such as TNF‐α, IL‐6, and IL‐12p70, which stimulate the Th1 response, were increased.^[^ ^8^ ^]^ Overall, the DA‐L‐DSA demonstrated efficient immune stimulation via maturation and migration into lymph nodes of DCs, and T cell activation compared to other groups.

Next, we considered the route of administration to effectively deliver DA‐L‐DSA to the dendritic cells. Focusing on the fact that many dendritic cells are distributed subcutaneously, the subcutaneous injection was chosen to be a proper administration route in vivo. To determine whether DA‐L‐DSA can be effectively delivered to dendritic cells to induce maturation and migration to lymph nodes, Cy5.5‐loaded DA‐L‐DSAs were injected subcutaneously into the mouse. Ex vivo fluorescence imaging and lymph node analysis confirmed that DA‐L‐DSA was selectively delivered to the dendritic cells and induced migration to the draining lymph nodes.

Based on these verifications, the effectiveness of anti‐tumor and anti‐metastatic immunotherapy through tumor animal modeling using cancer cells expressing survivin was investigated in two separate tumor models. B16F10 cells were utilized for skin cancer modeling in C57BL/6 mice for anti‐tumor efficacy and 4T1 cells were utilized for metastatic breast cancer modeling in Balb/c mice for anti‐metastatic efficacy. The therapeutic effectiveness of DA‐L‐DSA verified in two animal models represented significantly higher anti‐tumor and anti‐metastatic effects than other groups with adjuvants alone or with DA‐DSA without a lipid membrane. Both the maturation of dendritic cells within the lymph nodes and the distribution of T cells within the primary tumor were observed to effectively increase in the DA‐L‐DSA treatment group. These results confirm that without the survivin epitope, the adaptive immune response does not occur properly, and the lipid membrane of the nano‐assembly provides stability in the body, enhancing the ability to deliver adjuvants and the survivin peptide epitope to dendritic cells, resulting in effective anti‐tumor effects. As the influx of CTL into the primary tumor was also found to increase with DA‐L‐DSA treatment, the combination therapy of DA‐L‐DSA with immune checkpoint inhibitors was considered to synergistically activate immune response. Additional administration with α‐PD‐1 was verified in the B16F10 allograft melanoma model to most synergistically boost the anti‐tumor effect of DA‐L‐DSA compared to a single treatment with DA‐L‐DSA or α‐PD‐1.

Antigens expressed in cancer cells, which are recognized by T cells in our body, can be classified into tumor‐associated antigen (TAA) and tumor‐specific antigen (TSA). TAA is an antigen that appears more in cancer cells than in normal cells, which is not cancer cell specific. Therefore, the immune response using this is likely to be eliminated by immunotolerance or conversely, unwanted organs may be attacked due to autoimmunity. In contrast, TSA is an antigen specifically present only in cancer cells. This antigen generates a new antigen called neoantigen due to the mutation of the DNA portion. Since mutations do not occur in normal tissues, neoantigen‐specific T cells are free from immunotolerance or autoimmunity problems. Because of these advantages, neoantigens are considered an ideal target for T‐cell‐based cancer immunotherapy.^[^ ^7^, ^8^ ^]^ In this study, we have engineered a self‐assembling peptide antigen nano‐complex endowed with a dual‐adjuvant core, positioned as an innovative delivery system for anticancer peptide vaccines. Utilizing survivin peptide, a notable tumor‐associated antigen, marks a significant advancement in the targeted delivery of therapeutic agents in cancer immunotherapy. However, the burgeoning field of gene sequencing technology, particularly in the production of neoantigens, requires personalized cancer vaccines. By tailoring the peptide sequence within our nano‐complex to incorporate patient‐specific neoantigen epitopes, it becomes possible to craft highly individualized therapeutic interventions. Such personalized vaccines stand to revolutionize cancer treatment by directly addressing the heterogeneity of tumor antigens, thereby circumventing the limitations associated with low reactivity to immune checkpoint inhibitors and enhancing the efficacy of immunotherapeutic responses. The integration of neoantigen‐specific peptides within the dual‐adjuvant delivery platform could thus represent a potential strategy in the quest to overcome current challenges in cancer immunotherapy, offering a robust and adaptive solution to the complexities of tumor‐induced immune evasion.

## 4 Conclusion

DA‐L‐DSA represents a novel self‐assembling peptide antigen nano‐complex specifically designed to elicit an immune response against survivin, a tumor‐associated antigen. This complex incorporates a survivin‐derived peptide epitope that binds to the MHC class I molecules on dendritic cells, along with dual adjuvants and structural lipid membranes, to enhance immune system engagement and response. DA‐L‐DSA was injected subcutaneously into the murine allograft melanoma model, and the spontaneous breast cancer model was established using B16F10 cells and 4T1 cells which are characterized by survivin expression. DA‐L‐DSA was effectively delivered to dendritic cells to induce dendritic cell maturation, which in turn triggered the activation of naïve T cells by inducing their migration to lymph nodes, thereby promoting the influx of CTL into the tumor microenvironment. Consequently, DA‐L‐DSA monotherapy produced potent anti‐tumor and anti‐metastatic effects, and combined treatment with immune checkpoint blockade produced a synergistic effect, suggesting new opportunities for tumor‐associated antigen‐directed cancer immunotherapy.

## 5 Experimental Section

### Materials

The deoxycholic acid‐survivin_(66‐74)_ (DS, DCA‐Gly‐Trp‐Glu‐Pro‐Asp‐Asp‐Asn‐Pro‐Ile) was synthesized from GL Biochem Ltd. (Shanghai, China). 1,2‐Dipalmitoyl‐sn‐glycero‐3‐phosphocholine (DPPC) and 1,2‐distearoyl‐sn‐glycero‐3‐phosphoethanolamine‐N‐[amino(polyethylene glycol)−2000] (DSPE‐PEG_2000_) were purchased from Avanti Polar Lipids, Inc. (Alabaster, AL, USA). R848, SD‐208, Pyrene, Cy5.5, and CCK‐8 assay kit were obtained from Sigma‐Aldrich (St. Louis, MO, USA). Fetal bovine serum (FBS), Dulbecco's modified Eagle's medium (DMEM), and RPMI1640 medium were purchased from WELGENE (Seoul, Korea). Mouse recombinant GM‐CSF, IL‐4, DNase I solution, and collagenase/hyaluronidase were obtained from STEMCELL Technologies (Vancouver, Canada). Anti‐mouse CD80, CD40, CD86, CCR7, MHC I, MHC II, CD3, CD8, and IFN‐γ antibodies were obtained from Biolegend (San Diego, CA, USA). Anti‐mouse CD45 antibody was purchased from BD biosciences (Franklin Lakes, NJ, USA). β‐mercaptoethanol, Hanks’ balanced salt solution (HBSS), and mouse ELISA kits (IL‐12p70, IL‐6, TNF‐α) were obtained from Thermofisher Scientific (Waltham, MA, USA). DeadEnd fluorometric TUNEL system was obtained from Promega. (Wisconsin‐Madison, USA)

### Determination of Critical Micelle Concentration (CMC) of DS

To identify the CMC of DS, various concentrations of the DS (0 to 500 µg mL^−1^) were incubated with pyrene solution (25 ng mL^−1^) for 1 h. The fluorescence intensity of each solution (excitation at 330 nm) was measured with a plate reader. The ratios between fluorescence intensities measured at 373 and 384 nm were utilized to determine the critical micelle concentration (CMC) of the DS.

### Preparation of Lipid‐Coated DCA‐Survivin Assembly Containing Dual Adjuvants (DA‐L‐DSA)

The mixture of DSPE‐PEG_2000_ and DPPC was dissolved in chloroform at a molar ratio of 1:3 and stirred for 30 min at room temperature to evaporate. Then, the prepared lipid mixture was hydrated in water (4% EtOH, 10 mL) at 0.2 mg mL^−1^ and gently stirred. DS (1 mg), R848 (0.15 mg), and SD‐208 (0.15 mg) were dissolved in dichloromethane (1 mg mL^−1^). The premixed adjuvants/DS solution was dropwise slowly to a lipid solution (1.25 mL), sonicated, and evaporated to remove the organic solvent.

### Physicochemical Characterization of DA‐L‐DSA

Hydrodynamic size, zeta potential, and polydispersity index (PDI) of the DA‐L‐DSA were measured with Zetasizer‐Nano ZS (Malvern Instrument, Worcestershire, UK) to optimize lipid/DCA‐survivin, adjuvants/DCA‐survivin assembly (DSA) ratios. The morphological characteristic of the DA‐L‐DSA was visualized by transmission electron microscopy.

### Drug Loading and In Vitro Release Profiling

DA‐L‐DSA (0.25 mg) was used to calculate the drug loading and encapsulation efficiency of R848 and SD‐208 at absorbance 320 and 370 nm, respectively. The 1 mg mL^−1^ DA‐L‐DSA was resuspended in PBS (w or w/o serum 10%) and the released medium at 2, 4, 6, 12, 24, 48, 72, and 120 h were harvested through centrifugation. Release media and nanoassembly were lyophilized and resuspended to detect R848 and SD‐208 at absorbance 320 and 370 nm, respectively.

### Cell Culture

To obtain mouse bone marrow‐derived dendritic cells (BMDCs), bone marrow cells were collected from the femur and tibia of 6–10 week old C57BL/6 mice (Orient Bio, Korea). The contents of bone marrow were flushed with HBSS. The harvested cells were washed twice with HBSS and incubated at 37 °C for 6 d in RPMI1640 supplemented with 1% penicillin–streptomycin(100 U mL^−1^), 10% FBS, IL‐4 (20 ng mL^−1^), GM‐CSF (20 ng mL^−1^), β‐mercaptoethanol (55 × 10^−9^ m). On day 6, the cells were sorted using a magnetic EasySep Mouse CD11c Positive Selection Kit (STEMCELL Technologies, USA).

B16F10 (murine melanoma cell line) was obtained from ATCC (Virginia, USA). DMEM supplemented with 1% penicillin–streptomycin (100 U mL^−1^), 10 % FBS was used for B16F10 cell culture. The cells were incubated at 37 °C in 5 % CO_2_ atmosphere.

### In Vitro Cell Viability Test

Mouse bone marrow‐derived dendritic cells (BMDCs) were seeded in 96‐well plates and incubated for 24 h at 37 °C. Then the DA‐L‐DSA and other nanoassembly groups were added to BMDCs with a concentration of 0 × 10^−6^ to 20 × 10^−6^ m for 24 h, and the cells were incubated with CCK‐8 solution for 30 min. Then, the relative cell viability level to the control group was measured by UV/Vis spectrophotometer (Tecan) at 450 nm.

### Cellular Uptake and Confocal Microscopy Imaging

BMDCs (2 × 10^5^ cells mL^−1^) were incubated with Cy5.5‐loaded nano‐assembly at a concentration of 1.5 µg mL^−1^ for 2 h and analyzed using FACS Calibur (BD Biosciences, USA). Cells were stained with DAPI (Southern Biotech) and visualized by confocal microscopy (Leica).

### Measurement of In Vitro Dendritic Cell Maturation

BMDCs (2 × 10^5^ cells mL^−1^) were incubated with nanoassembly at a concentration of 1.5 µg mL^−1^ for 24 h. Then cells were stained with anti‐mouse CD80, anti‐mouse CD86, anti‐mouse CD40, anti‐mouse MHCI, anti‐mouse MHCII, and anti‐mouse CCR7. After the cells were washed twice and resuspended in ice‐cold PBS (10% FBS), they were analyzed using FACS Calibur (BD Biosciences, USA).

### Western Blot

Cells were lysed in RIPA buffer with a protease inhibitor cocktail and phosphatase inhibitor cocktail (Sigma‐Aldrich) and centrifuged. Total cell lysates were resuspended in the SDS sample buffer and resolved using SDS‐PAGE. Proteins were transferred to Immobilon‐P PVDF membrane(Sigma‐Aldrich) and blocked with 5% bovine serum albumin (BSA)/TBST buffer for 1 h at room temperature. Membranes were incubated with primary antibodies overnight. Primary antibodies used for western blotting were anti‐mouse CD80 (1:1000, Abcam, Boston, USA), anti‐mouse CD86 (1:1000, Cell Signaling, Massachusetts, USA), anti‐mouse, and mouse anti‐GAPDH (1:1000, Abcam). Next, membranes were incubated with secondary antibodies for 1 h at room temperature. Anti‐rabbit HRP‐linked antibody (1:2000, Cell Signaling) was used as the secondary antibody. The membranes were captured using ChemiDoc XRS+ (Bio‐Rad, California, USA).

### Measurement of In Vitro Cytokine Level

BMDCs (2 × 10^5^ cells mL^−1^) were incubated with the DA‐L‐DSA and other nano‐assembly groups at a concentration of 1.5 µg mL^−1^ for 24 h. Then, culture media supernatant from each group was obtained and centrifuged at 13 000 rpm for 5 min at 4 °C. The protein expression levels of IL‐12p70, IL‐6, and TNF‐α in the supernatant samples were quantified by using ELISA kits (Invitrogen, USA).

### In Vivo Biodistribution Test

After subcutaneous injection of Cy5.5‐loaded nano‐assembly into mice, the liver, spleen, heart, lung, and inguinal lymph nodes were harvested 24 h later for fluorescence signal analysis. The isolated major organs were analyzed using the FOBI device.

### In Vivo Cytotoxic T Lymphocyte Antigen‐Specific Killing Assay

C57BL/6 mice were administered subcutaneously with 20 µg of DS (DCA‐survivin_(66‐74)_ conjugate), and different experimental groups were constructed based on the drug loading (%) of DA‐L‐DSA every 5 d for three times, respectively. 7 d after the final vaccination, splenocytes of untreated healthy mice were collected and treated with 1 µg of surviving peptides or PBS about 4 h. Then, the surviving treated splenocytes and PBS‐treated splenocytes were labeled using carboxyfluorescein succinimidyl ester (CFSE) with 5 µM and 0.5 µM, respectively. Afterward, each splenocytes were mixed with the same numbers, and injected intravenously into each immunized mouse. After 18 h, the percentages of CFSE^[high]^ and CFSE^[low]^ in spleen of each mouse were detected via flow cytometry for calculating the percentages of antigen‐specific killing ability by the following formula:

(1) $$\mathrm{Specific}\;\mathrm{killing}\;\left(\%\right)=\left[1-\left(\mathrm{CFS}E^{\left[\mathrm{high}\right]}/\mathrm{CFS}E^{\left[\mathrm{low}\right]}\right)\right]\times 100\%$$

### In Vivo Therapeutic Effects on Murine Melanoma Model and Breast Cancer Model

A murine melanoma model was established with 6 to 10 week old C57BL/6 mice inoculating 2 × 10^5^ B16F10 cells by subcutaneous injection into the left flank. A spontaneous metastatic breast cancer model was established with 6 to 10 week old Balb/c mice. Mice were inoculated with 2 × 10^5^ 4T1 cells by subcutaneous injection into the 4^th^ mammary gland. The nano‐assembly, including 20 µg of survivin was injected by subcutaneous injection into the right flank, and tumor size (mm^3^) was calculated by (length) × (width)^2^ × 1/2. The experiment was stopped on day 16. In the case of breast cancer model, surgical resection was performed on day 16 to further observe the anti‐metastatic effect, and the lungs with metastasis were separated and observed on day 30.

All animal experiments were conducted according to the protocol approved by the Institutional Animal Care and Use Committee of Hanyang University, registered as 2022‐0051A.

### Analysis of Tumor‐Infiltrating Lymphocytes (TIL) in Primary Tumor

The mice from each group were sacrificed to analyze tumor‐infiltrating lymphocytes on day 20. The tumor tissues were harvested and digested with DNase I solution, collagenase/hyaluronidase. The digested tumor tissues were filtered through a 70 µm strainer and centrifuged at 300 *g* for 10 min at room temperature. RBCs were lysed, and the cells were washed twice with PBS. Then, cells were fixed with 4% paraformaldehyde (Wako, Japan) for 15 min, permeabilized with cell permeabilization buffer (Thermofisher Scientific, USA) for 10 min at room temperature, and stained with anti‐mouse CD45, anti‐mouse CD3, anti‐mouse CD8, anti‐mouse IFN‐γ. After the cells were washed twice and resuspended in ice‐cold PBS supplemented with 10% FBS, they were analyzed using FACS Calibur (BD Biosciences, USA). For the immunofluorescence study, the paraffin sections of tumor tissues were prepared. They were deparaffinized, permeabilized, and stained with anti‐mouse CD3, and anti‐mouse CD8. After the section was washed twice, the cell nuclei were stained with DAPI solution. In addition, the tumor cells were stained by following the protocol using DeadEnd Fluorometric TUNEL System for detecting apoptosis. The slides were examined under a fluorescence microscope.

### Figure Images

The illustrations of the synthetic procedure and mechanism of action of DA‐L‐DSA, as depicted in Figure 1, were produced with the assistance of Biomed Art (Korea). Furthermore, the animal images incorporated into Figures 5, 6, 7 were generated through the use of the Biorender.

### Statistical Analysis

All data were represented as the mean ± SD and SEM. Statistical analyses were performed using a Student's t‐test and one‐way ANOVA with Tukey's post‐hoc test in GraphPad Prism 8 Project software for Windows (GraphPad Software). All *p*‐values were considered statistically significant (*p < 0.05, **p < 0.01, ***p < 0.001, ns = nonsignificant). Detailed processing of data and sample size for each analysis are described in the figure legends.

## Conflict of Interest

The authors declare no conflict of interest.

## Supporting information

Supporting Information

## Data Availability

The data that support the findings of this study are available from the corresponding author upon reasonable request.

## References

[1] A. D. Waldman , J. M. Fritz , M. J. Lenardo , Nat. Rev. Immunol. 2020, 20, 651. 32433532 10.1038/s41577-020-0306-5 PMC7238960

[2] S. K. Wculek , F. J. Cueto , A. M. Mujal , I. Melero , M. F. Krummel , D. Sancho , Nat. Rev. Immunol. 2020, 20, 7. 31467405 10.1038/s41577-019-0210-z

[3] I. Heras‐Murillo , I. Adán‐Barrientos , M. Galán , S. K. Wculek , D. Sancho , Nat. Rev. Clin. Oncol. 2024, 21, 257. 38326563 10.1038/s41571-024-00859-1

[4] P. W. Kantoff , C. S. Higano , N. D. Shore , E. R. Berger , E. J. Small , D. F. Penson , C. H. Redfern , A. C. Ferrari , R. Dreicer , R. B. Sims , Y. Xu , M. W. Frohlich , P. F. Schellhammer , T. Ahmed , A. Amin , J. Arseneau , N. Barth , G. Bernstein , B. Bracken , P. Burch , V. Caggiano , J. Chin , G. Chodak , F. Chu , J. Corman , B. Curti , N. Dawson , J. F. Deeken , T. Dubernet , M. Fishman , et al., New Engl. J. Med. 2010, 363, 411. 20818862 10.1056/NEJMoa1001294

[5] M. J. Lin , J. Svensson‐Arvelund , G. S. Lubitz , A. Marabelle , I. Melero , B. D. Brown , J. D. Brody , Nat. Cancer. 2022, 3, 911. 35999309 10.1038/s43018-022-00418-6

[6] W. S. Liu , H. C. Tang , L. F. Li , X. Y. Wang , Z. J. Yu , J. P. Li , Cell Proliferation 2021, 54, e13025. 33754407 10.1111/cpr.13025 PMC8088465

[7] H. Dong , Q. Li , Y. Zhang , M. Ding , Z. Teng , Y. Mou , Adv. Sci. 2023, 10, e2301339. 10.1002/advs.202301339 PMC10288267 37088780

[8] M. Saxena , S. H. van der Burg , C. J. M. Melief , N. Bhardwaj , Nat. Rev. Cancer 2021, 21, 360. 33907315 10.1038/s41568-021-00346-0

[9] D. C. Altieri , Nat. Rev. Cancer 2008, 8, 61. 18075512 10.1038/nrc2293

[10] a) S. R. Burkholz , C. V. Herst , R. T. Carback , P. E. Harris , R. M. Rubsamen , Vaccines 2023, 11, 644; 36992227 10.3390/vaccines11030644 PMC10051918

[11] R. D. Weeratna , S. R. Makinen , M. J. McCluskie , H. L. Davis , Vaccine 2005, 23, 5263. 16081189 10.1016/j.vaccine.2005.06.024

[12] S. M. Jin , Y. J. Yoo , H. S. Shin , S. Kim , S. N. Lee , C. H. Lee , H. Kim , J. E. Kim , Y. S. Bae , J. Hong , Y. W. Noh , Y. T. Lim , Nat. Nanotechnol. 2023, 18, 390. 36635335 10.1038/s41565-022-01296-w

[13] a) J. Kim , M. Kim , S. B. Yong , H. Han , S. Kang , S. F. Lahiji , S. Kim , J. Hong , Y. Seo , Y. H. Kim , Biomater. Res. 2023, 27, 136; 38111068 10.1186/s40824-023-00470-y PMC10729390

[14] a) J. J. Kobie , R. S. Wu , R. A. Kurt , S. M. Lou , M. K. Adelman , L. J. Whitesell , L. V. Ramanathapuram , C. L. Arteaga , E. T. Akporiaye , Cancer Res. 2003, 63, 1860; 12702574

[15] a) L. N. Wei , Y. Zhang , R. X. Wang , S. Liu , J. Luo , Y. F. Ma , H. Wang , Y. Liu , Y. Chen , Biomaterials 2023, 302, 122297; 37666102 10.1016/j.biomaterials.2023.122297

[16] a) J. Kim , J. Hong , J. Lee , S. F. Lahiji , Y. H. Kim , J. Controlled Release 2021, 332, 109; 10.1016/j.jconrel.2021.02.002 33571549

[17] S. W. VanGool , P. Vandenberghe , M. DeBoer , J. L. Ceuppens , Immunol. Rev. 1996, 153, 47. 9010719 10.1111/j.1600-065x.1996.tb00920.x

[18] M. H. Deng , T. Ma , Z. Z. Yan , K. R. Zettel , M. J. Scott , H. Liao , A. Frank , A. E. Morelli , C. P. Sodhi , D. J. Hackam , T. R. Billiar , J. Infect. Dis. 2016, 213, 1280. 26603204 10.1093/infdis/jiv562 PMC4799664

[19] E. P. Brandum , A. S. Jorgensen , M. M. Rosenkilde , G. M. Hjorto , Int. J. Mol. Sci. 2021, 22, 8340. 34361107 10.3390/ijms22158340 PMC8348795

