# Supplementary material for: Dual Adjuvant‐Loaded Peptide Antigen Self‐Assembly Potentiates Dendritic Cell‐Mediated Tumor Immunotherapy
Source: Adv Sci (Weinh). 2024 Jul 29;11(36):2403663. doi: 10.1002/advs.202403663 (PMC11423174; doi:10.1002/advs.202403663)
Supplement: Supplementary file 1 — Supporting Information [file ADVS-11-2403663-s001.docx]

Supporting Information

**Hydrophobized peptide antigen self-assembly with dual-adjuvant core potentiates dendritic cell-mediated tumor immunotherapy**

*Jaehyun Kim^1^, Seyoung Kang^1^, Jisoo Kim^1^, Seok-Beom Yong^2^,Shayan Fakhraei Lahiji^1,4^ and Yong-Hee Kim^1,3,4^**

^1^ Department of Bioengineering, Institute for Bioengineering and Biopharmaceutical Research

Hanyang University, 04763, Seoul, Republic of Korea

^2^ Nucleic Acid Therapeutics Research Center, Korea Research Institute of Bioscience and Biotechnology (KRIBB), Chungcheongbuk-do 28116, Republic of Korea

^3^ Institute for Bioengineering and Biopharmaceutical Research (IBBR), Hanyang University, 04763, Seoul, Republic of Korea

^4^ CURSUS BIO Inc. Icure Tower, Seoul, 06170, Republic of Korea

*Correspondence: Yong-Hee Kim, Department of Bioengineering, Hanyang University, Seoul, Republic of Korea

E-mail: yongheekim@hanyang.ac.kr

**Supporting Figures**

**Figure S1. The mass spectrometry results of deoxycholic acid-survivin_(66-74)_ conjugate (DS)**

The deoxycholic acid-survivin_(66-74)_conjugate (DS, DCA-Gly-Trp-Glu-Pro-Asp-Asp-Asn-Pro-Ile) represented a molecular weight of about 1,415Da, indicating that the synthesis of DS was made as expected.

**
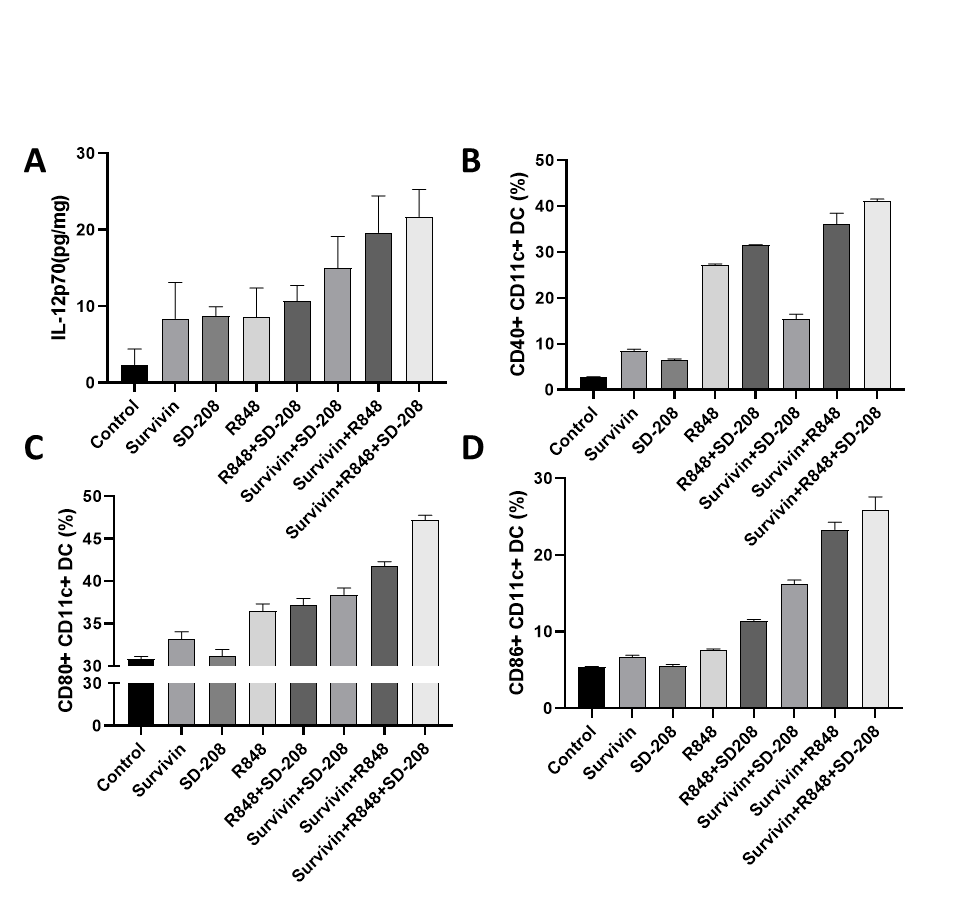
**

**Figure S2. R848 and SD-208 effectively induced dendritic cell maturation.**

R848 and SD-208-treated bone marrow-derived dendritic cells were analyzed. A) Enzyme-linked immunosorbent assay (ELISA). The extracellular secretion of IL-12p70 from bone marrow-derived dendritic cells (BMDCs) was measured. B-D) Flow cytometric analysis of surface expression of CD40, CD80, and CD86. ELISA and flow cytometric analysis confirmed that the combined treatment of R848 and SD-208 effectively induced dendritic cell maturation.

**
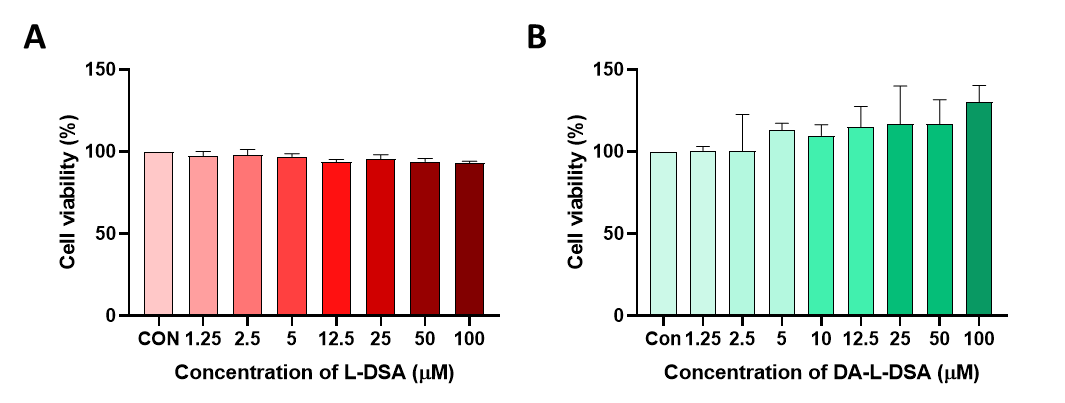
**

**Figure S3. In vitro cytotoxicity of L-DSA and DA-L-DSA**

In vitro cytotoxicity of L-DSA and DA-L-DSA was measured by CCK-8 assay with BMDCs incubated at various concentrations. Neither L-DSA nor DA-L-DSA exhibited cytotoxicity at concentrations up to 100μM.

**
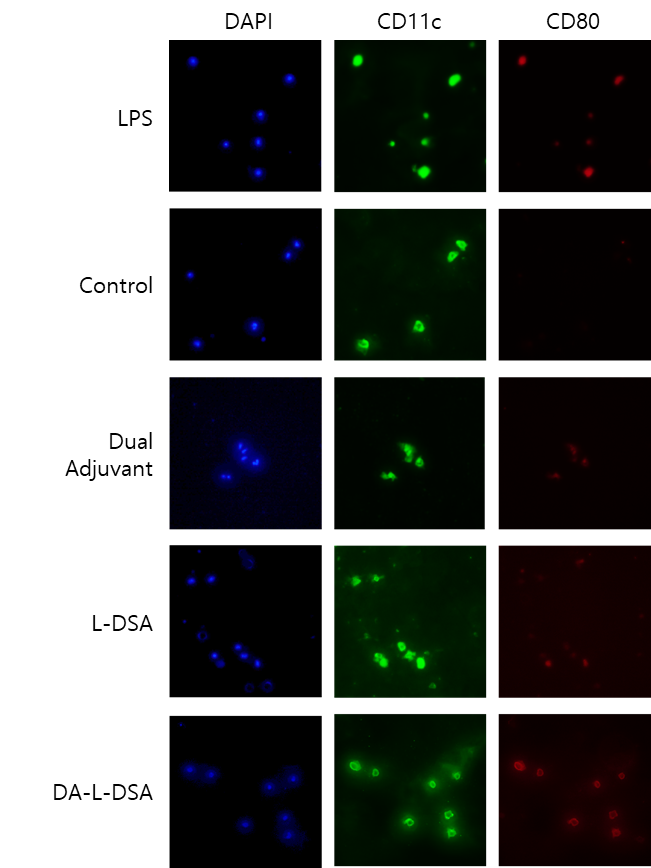
**

**Figure S4. In vitro evaluation of dendritic cell maturation with DA-L-DSA by fluorescence microscopy**

To determine whether DA-L-DSA can mature dendritic cells effectively and induce T cell activation, CD80 on the surface of the BMDCs was observed by fluorescence staining. The DA-L-DSA-treated group showed a similar level of CD80 expression to the LPS-treated group, which is a positive control. Green = CD11c, Red = CD80, Blue = DAPI.


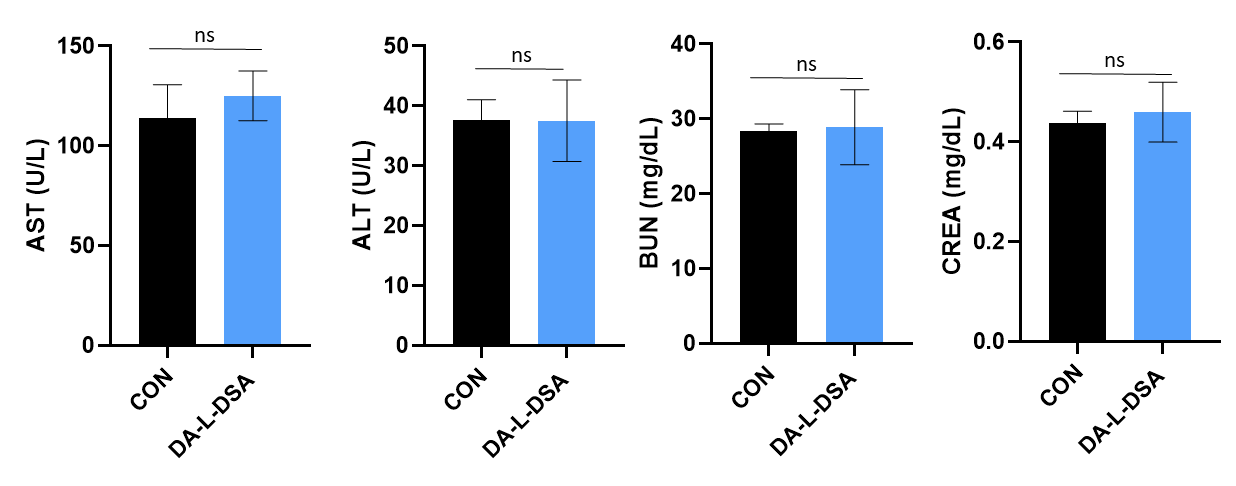


**Figure S5. In vivo toxicity evaluation of DA-L-DSA**

Toxicity was analyzed by measuring the levels of enzymes reflecting the functions of the liver and kidneys, such as aspartate aminotransferase (AST), alanine aminotransferase (ALT), blood urea nitrogen (BUN), and creatinine (CREA) in the plasma of 4T1 tumor-bearing mice on the day of last injection. Data represent mean ± SD. ns = not significant difference. Statistical analysis was calculated by One-Way ANOVA with Tukey’s post hoc test.

**
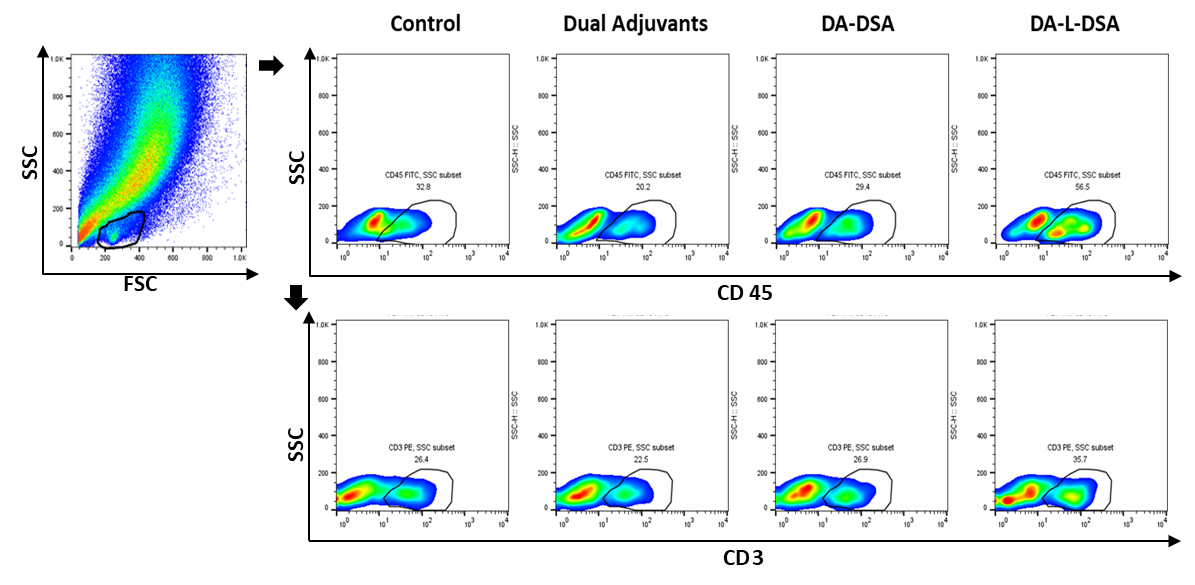
**

**Figure S6**. **Gating strategy of flow cytometric analysis for tumor-infiltrating lymphocytes from primary tumors**

The tumor tissues were separated into single-cell units. In addition, single cells were selected as far as possible from CD45-expressing cells using a magnetic separation assay, and regions of CD45^+^ and CD3^+^ expression were observed.

**
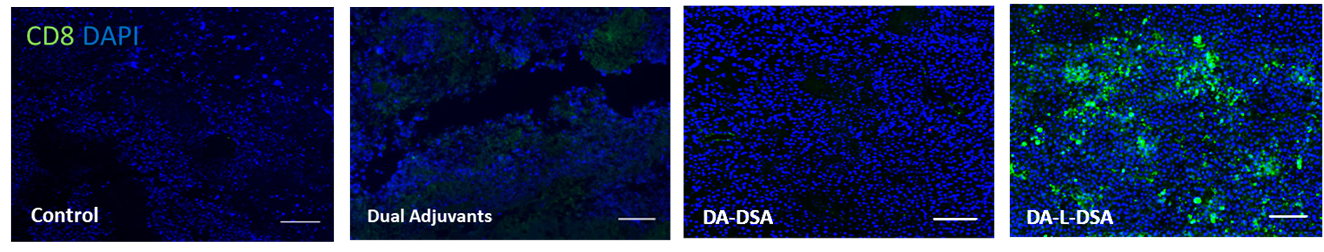
**

**Figure S7. Immunofluorescence staining image of the primary tumor**

Representative immunofluorescence staining image of CD8^+^ T cells in the tumor. DA-L-DSA exhibited significant recruitment of CD8 ^+^ T cells. Blue=DAPI, Green=CD8.

**
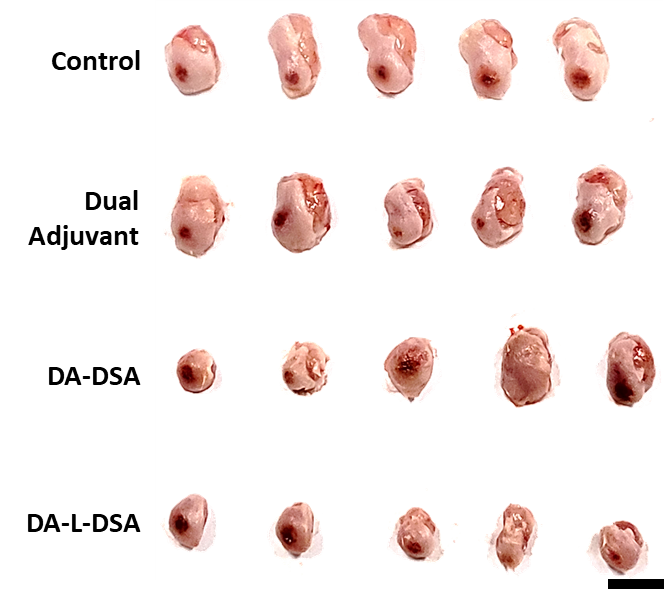
**

**Figure S8. Tumor images Isolated from metastatic breast cancer model**

To observe the therapeutic effect of a metastatic breast cancer model, DA-L-DSA and the other group were treated three times at 3-day intervals. On day 16, the tumor was removed by surgical resection. The scale bar represents 5mm.


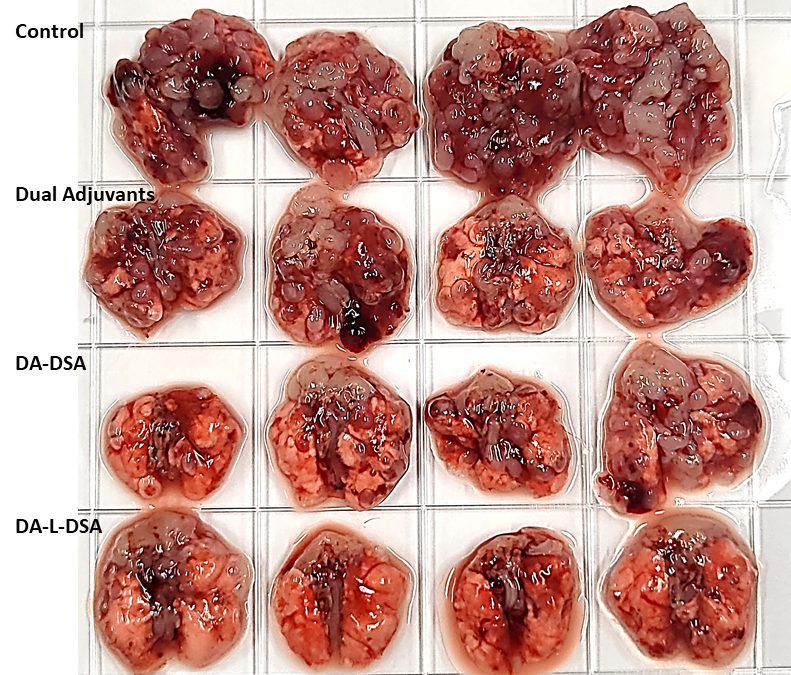


**Figure S9. Lung images isolated from metastatic breast cancer model**

To observe anti-metastatic effect of DA-L-DSA, lungs were isolated on Day 30. DA-L-DSA showed significantly reduced metastasis when compared with other comparison groups.


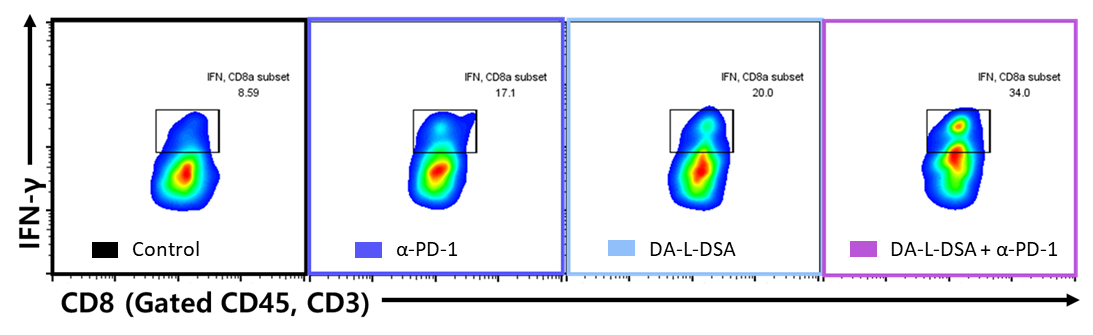


**Figure S10. Flow cytometric analysis of the primary tumors for verifying cytotoxic T cell activation**

To ascertain the efficacy of DA-L-DSA in enhancing the response rate of immune checkpoint inhibitors, the activity of cytotoxic T cells within tumors was evaluated through flow cytometric analysis. The results demonstrated a 1.98-fold increase in the anti-PD-1 alone group and a 1.7-fold increase in the DA-L-DSA alone group, respectively.
